# Clinical value of vestibular-evoked myogenic potential tests in patients with sudden sensorineural hearing loss

**DOI:** 10.1186/s12883-019-1576-z

**Published:** 2019-12-21

**Authors:** Yuan Wang, Shun-Tong Gu, Xiao-Lin Bao, Jia-Liang Guo

**Affiliations:** 1 Department of Otolaryngology-Head & Neck Surgery, Tianjin TEDA Hospital, Tianjin, China; 2 Department of General Surgery, Tianjin Fifth Central Hospital, Tianjin, China

**Keywords:** Sudden sensorineural hearing loss, Vertigo, Vestibular evoked myogenic

## Abstract

**Background:**

This study aims to investigate the clinical value of two kinds of vestibular-evoked myogenic potentials in patients with sudden sensorineural hearing loss (SSNHL).

**Methods:**

A total of 82 patients were divided into two groups: vertigo group and non-vertigo group. All patients underwent examinations for pure tone hearing thresholds, middle ear analysis, the videonystagmography, caloric tests, and vestibular-evoked myogenic potentials elicited from the sternocleidomastoid and extraocular muscle. In addition, 30 healthy subjects were selected as the control group.

**Results:**

For the 30 healthy subjects, the average latency of p13 and n23 of the cervical vestibular evoked myogenic potentials (cVEMPs) were 13.13 ± 2.89 ms and 23.51 ± 3.25 ms, respectively, and the bilateral amplitude asymmetry rate ranged within 0.05–0.31. The average latency of n10 of the ocular vestibular evoked myogenic potentials (oVEMPs) was 10.13 ± 0.48 ms. The average amplitude of the n10-p15-wave was 5.58 ± 0.65 μV. Among the 35 vertigo patients with SSNHL, 27 patients had normal cVEMP and oVEMP examination results, five patients had abnormal oVEMP examination results, and five patients had abnormal cVEMP examination results. The latency and amplifier of oVEMPs and cVEMPs were within the normal range in 47 SSNHL patients without vertigo. The chi-square value was 5.647, the *P*-value was equal to 0.017, and the difference was statistically significant at a confidence interval of 95%.

**Conclusions:**

OVEMPs and cVEMPs can be used evaluate the vestibular nerve function of SSNHL patients with vertigo.

## Background

Sudden sensorineural hearing loss (SSNHL), namely sudden deafness, is a common disease of the ear, nose and throat (ENT), which mostly happens unilaterally, and sensorineural hearing loss with unknown cause (i.e. idiopathic), manifesting as different degrees of severity up to complete deafness. The Otolaryngology Head and Neck Surgery Branch of the Chinese Medical Association revised the diagnostic criteria for sudden hearing loss in 2015 [1]: SSNHL onset may occur without apparent cause, at least in the adjacent two frequency hearing loss of ≥20 dBHL, and reaches its peak in 72 h. Vertigo and/or tinnitus may additionally appear [2]. Although vascular, viral, or multiple etiologies have been proposed as causes of SSNHL, up to 90% of SSNHL cases are considered idiopathic [3]. Vertigo may develop in approximately 30–60% of patients with SSNHL [4]. SSNHL accompanied with vertigo is usually considered to be caused by vestibular involvement. The vestibular-evoked myogenic potential (VEMP) is formed by myogenic responses activated by acoustic, galvanic, or bone vibration stimulation, and this is recorded by surface electromyography [5]. Cervical VEMP (cVEMP) was first introduced by Colebatch et al. The aim of this method is to assess the myogenic activity of the sternocleidomastoid muscle (SCM) [6]. Up to its advent, it was impossible to evaluate the otolithic organs [7–10].

In recent years, studies have shown that the cVEMP response to balloon and vestibular nerve function, while ocular VEMPs (oVEMPs) can respond to the upper vestibular nerve. The combination of these two tests may be able to respond to the vestibular nerves function in patients with SSNHL. Hence, the aim of this study was to evaluate the clinical value of two kinds of vestibular-evoked myogenic potentials in patients with SSNHL. The results are presented and summarized as follows.

## Methods

### Subjects

According to the diagnostic criteria for SSNHL, the Chinese Journal of Otorhinolaryngology Head and Neck Surgery in 2015, and the vertigo symptoms, a total of 82 SSNHL patients were divided into two groups. Patients with vertigo were assigned to the vertigo group, while patients without vertigo were assigned to the non-vertigo group. All patients in the vertigo group exhibited nystagmus and the pattern of nystagmus did not change during the disease course. Patients with relapsed vertigo were excluded. All patients were unilateral and had no diseases, such as benign paroxysmal positional vertigo, labyrinthitis, vestibular neuritis, drug toxicity vertigo, vertebrobasilar insufficiency, and intracranial space-occupying lesions. A total of 30 healthy subjects with normal hearing and without vertigo were selected and assigned as the control group.

All subjects underwent the necessary physical examination, neurological examination, necessary laboratory test, and temporal bone computed tomography (CT) scan. And the pure tone hearing threshold, middle ear analysis, video nystagmography, caloric test data, cVEMPs and oVEMPs were also obtained.

### Caloric testing

Caloric testing was performed by irrigating the external auditory canal with 2 ml ice water (4 °C) for 20 s followed by aspiration of water. Caloric nystagmus was recorded, in a darkened room, using an electronystagmograph. We defined an abnormal caloric response by either of the following criteria: (1) canal paresis (CP) percentage > 20%; (2) maximum slow phase eye velocity < 10/s bilaterally.

### Stimuli and recording techniques of cVEMPs [11]

A commercial electromyographic (EMG) system (MEB-9200 K, Japan) was used for the cVEMP test. Patients were tested in a sitting position during the cVEMP procedure. The disc-shaped recording electrode was placed at the midpoint of the sternocleidomastoid muscle (SMC) belly, a reference electrode was placed at the SMC clavicle, and a ground electrode was placed at the mid-forehead skin surface. Patients were asked to turn their heads contralaterally to keep the SCM muscle contracted. The skin surface impedance was < 5 kΩ. Using the air guide click sound stimulation, the intensity was 105 dBnHL, stimulation frequency was three times per second, bandpass filter range was 500–2000 Hz, and superimposition was 200 times. Sound stimuli were delivered monaurally and recorded bilaterally.

The latencies and amplitudes of the first positive–negative peaks (p13–n23) of the cVEMP were evaluated as described by Fujimoto et al. [12]. Briefly, the asymmetry ratio (AR) for p13–n23 amplitude (cVEMP AR) was calculated as 100 [(Au - Aa)/(Aa + Au)], where Au is the p13–n23 amplitude on the unaffected side and Aa is the p13–n23 amplitude on the affected side. On the basis of the results from normal subjects, the upper limit of the percentage cVEMP AR was placed. When no reproducible p13–n23 was present in 2 runs, we regarded it as an “absent response”. When a reproducible p13–n23 was present and cVEMP AR (%) was greater than the normal upper limits, we regarded it as a “decreased response”.

### Stimuli and recording techniques of oVEMPs [13]

A commercial EMG system (MEB-9200 K, Japan) was used for the oVEMP test. The patients were instructed to lie in the supine position. The standard was placed at 70 cm from the top of the head, with 25 degrees backward positioning. A disc-shaped recording electrode was placed at the middle of the lower side of the pupil at 10 mm. The reference electrode was placed at the skin at 20 mm lower than that of the recording electrode. The ground electrode was placed at the middle of the forehead. The skin surface impedance was < 5 kΩ. The air guide 500-Hz click sound stimulation was employed, the intensity was 105 dBnHL. Sound stimuli were monaurally delivered and bilaterally recorded. Stimulus frequency was three times per second, bandpass filter range was 20–500 Hz, and the superimposition was 50 times. The patients were prohibited from moving their head and instructed to keep their eyes on the visual test-object during the test. A significant negative waveform, named n10, was recorded at approximately 10 s after stimulus onset. The latency and amplitude of n10 were calculated. We analyzed the first negative peak (n10) latency, the subsequent positive peak (p15) latency, and the amplitude between n10 and p15. For the evaluation of amplitude, the asymmetry ratio for n10–p15 amplitude (oVEMP AR) was calculated as 100 [(Au - Aa)/(Aa + Au)], where Au is the n10–p15 amplitude on the unaffected side and Aa is the n10–p15 amplitude on the affected side. Responses recorded from the eye contralateral to stimulation were used for calculating oVEMP AR. On the basis of the results from normal subjects, the upper limit of normal oVEMP AR was set. When no reproducible n10–p15 was present in 2 runs, we regarded it as an “absent response”. When a reproducible n10–p15 was present and the oVEMP AR (%) was greater than the normal upper limits, we regarded it as a “decreased response” [12].

### Statistical analysis

For statistical analysis, SPSS 17.0 software was used. The statistical results were presented as mean ± standard deviation (SD). The chi-square was used to analyze the data between the two groups using the SPSS 17.0 software. The chi-square value was 5.647, the *P*-value was equal to 0.017, and the difference was statistically significant at a confidence interval of 95%.

## Results

Among the 35 patients with vertigo, 16 patients had SSNHL in the left ear, while 19 patients had SSNHL in the right ear. Furthermore, among these patients, 21 patients were male, while 14 patients were female, and the age of these patients ranged within 23–49 years old. Vertigo almost simultaneously occurred at onset, without other nerve involvement symptoms. And the vertigo continued from one hour to five days. In addition, 32 patients were accompanied by nausea, while 16 patients were accompanied by vomiting. Among the 47 patients without vertigo, 23 patients had SSNHL in the left ear, while 24 patients had SSNHL in the right ear. 26 patients were male and 21 patients were female. And the age of these patients ranged within 21–46 years old. Among the 30 healthy subjects, 16 subjects were male and 14 subjects were female. And the age of these healthy subjects ranged within 22–48 years old (Table 1).

**Table 1:** The characteristics of each group

Characteristics	Vertigo group (*n* = 35)	non-vertigo group (*n* = 47)	healthy subjects (*n* = 30)
Age	23–49 years	21–46 years	22–48 years
Sex			
Male	21	26	16
Female	14	21	14
Side			
Right	19	24	–
Left	16	23	–
Accompanying symptoms			
Nausea	32	–	–
Vomiting	16	–	–
Severity of deafness			
Mild	6	6	–
Moderate	21	29	–
Severe	5	8	–
Extremely severe	3	4	–
Caloric responses			
Normal	33	47	30
Abnormal	2	0	0

cVEMPs and oVEMPs were both evoked in the 30 healthy subjects. And the caloric tests were all normal. The latency of p13 and n23 of the cVEMP was 13.13 ± 2.89 ms and 23.51 ± 3.25 ms, respectively. The amplitude asymmetry ranged within 0.05–0.31. The latency of the n10 waveform of the oVEMP was 10.13 ± 0.48 ms, while the mean amplitude of the n10-p15 waveform was 5.58 ± 0.65 μV. The amplitude asymmetry between the two sides ranged within 0.05–0.35.

**Fig. 1:**
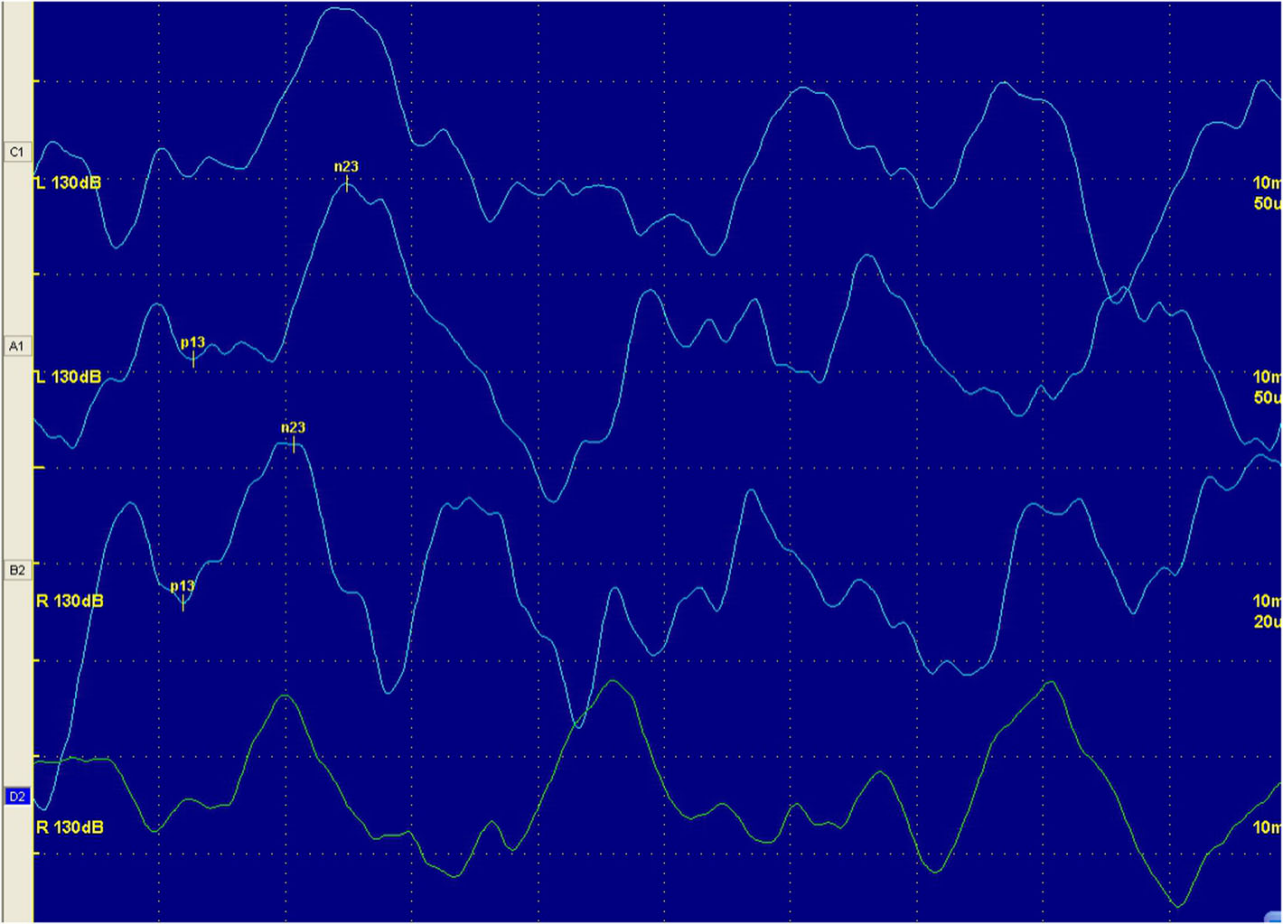
cVEMP of a 30-year-old man in vertigo group. This patient showed extented latency of left ear, but showed normal cVEMP on right ear

**Fig. 2:**
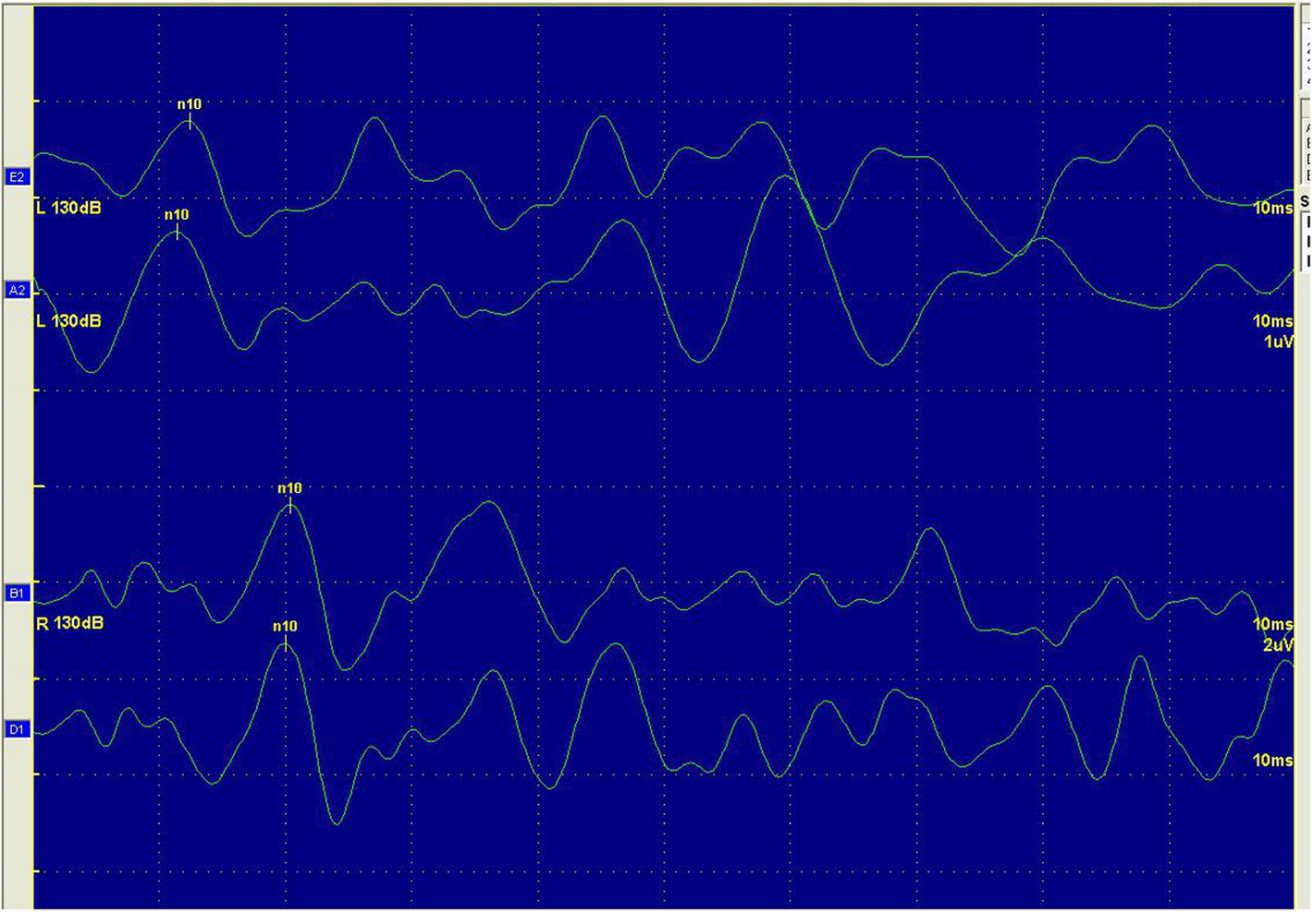
oVEMP of a 42-year-old woman in vertigo group. This patient showed extented latency of right ear, but showed normal oVEMP on left ear

Among the 35 SSNHL patients with vertigo, six patients had mild deafness, 21 patients had moderate deafness, five patients had severe deafness, and three patients had extremely severe deafness. 2 of the 35 SSNHL patients with vertigo showed abnormal caloric responses on the affected side. Furthermore, 30 patients had normal latency and amplitude of cVEMP and oVEMP, five patients had abnormal cVEMP (two patients had an extended p13 latency, one patient had an extended n23 latency, and one patient had extended p13 and n23 latencies), five patients had abnormal oVEMPs (three patients had a n10 extended latency, while two patients were not evoked). Figures 1 and 2 are representative cVEMP and oVEMP images in the vertigo group.

Among the SSNHL 47 patients without vertigo, six patients had mild deafness, 29 patients had moderate deafness, eight patients had severe deafness, and four patients had extremely severe deafness. All patients had normal caloric responses. The oVEMP and cVEMP waveforms and latency were in the normal range.

As shown in Table 2, abnormal results for cVEMP and oVEMP can be detected in 25.7% (5/35) of patients with vertigo, which was statistically significant, when compared to patients without vertigo (*p* = 0.017).

**Table 2:** Analysis of association by the Chi-squared test according to the tests of cVEMPs and oVEMPs

Tests	SSNHL with vertigo		SSNHL without vertigo		*X*^2^	P
	Abnormal	Normal	Abnormal	Normal		
cVEMPs	5	27	0	42	5.647	0.017
oVEMPs	5	27	0	42	5.647	0.017

## Discussion

SSNHL is a type of sensorineural deafness without apparent cause. The cause of SSNHL with vertigo is more complex, and clinical hearing loss is usually worse [14]. The probability of hearing recovery is also significantly lower, when compared to those without vertigo [15–17].

Studies have shown that SSNHL patients with vertigo are different from patients with Meniere’s disease. This is mostly caused by the involvement of vestibular nerve afferent receptors, in which the symptoms of vertigo rapidly improves through the rapid establishment of the vestibular central compensatory mechanism. The function of the vestibular nerve is essential for the diagnosis and differential diagnosis of SSNHL with vertigo.

The cVEMP can be used to assess the saccular function and inferior vestibular pathway, while oVEMP can be used to evaluate the utricular function and superior vestibular pathway [18–20]. For patients with SSNHL, the cVEMP and oVEMP tests can be more effective responses to vestibular nerve function [21].

In the present study, 82 patients with SSNHL underwent cVEMP and oVEMP tests. Abnormal results for cVEMP and oVEMP can be detected in 25.7% (9/35) of patients with vertigo, which was statistically significant, when compared to patients without vertigo. Hence, there is a certain clinical value to test the VEMP of SSNHL patients with vertigo. The abnormal rate of VEMP is high in patients with moderate to extremely severe deafness. Further observations are needed to determine the specificity of the diagnosis of vertigo. Moreover, more cases were needed to prove the significance of latency and amplitude before and after treatment.

From the abnormal rate of cVEMPs and oVEMPs, it can be concluded that the involvement of the upper vestibular nerve and lower vestibular nerve in SSNHL patients with vertigo were roughly similar, but the difference was not statistically significant. From the correlation analysis of cVEMPs and oVEMPs with pure tone and middle ear analysis, the chi-square test result was not statistically significant. Hence, it can be concluded that VEMP is not correlated to the degree of hearing loss and the functional status of the middle ear. Further studies are needed to determine whether VEMPs are correlated to cochlear electrograms, video electroencephalography, caloric tests and other vestibular function tests.

## Conclusion

Sudden deafness is common in ENT, but the pathogenesis of patients with vertigo is more complex. Patients are at a substantially increased risk of vestibular organ lesions following a diagnosis of SSNHL, especially in the presence of vertigo [22]. For the initial onset of vertigo patients, it remains difficult to identify SSNHL accompanied with vertigo or Meniere’s disease, which is caused by dizziness associated with hearing loss. Patients with SSNHL accompanied by vertigo have no specific findings with cVEMPs and oVEMPs tests. Hence, VEMP tests can provide evidence for the lesion location of the vestibular, and provide objective information for further clinical observation.

## Abbreviations

CP: canal paresis
CT: computed tomography
cVEMP: cervical VEMP
EMG: electromyographic
ENT: ear, nose and throat
oVEMPs: ocular VEMPs
SCM: sternocleidomastoid muscle
SD: standard deviation
SSNHL: sudden sensorineural hearing loss
VEMP: vestibular-evoked myogenic potential
